# Editorial: Precision medicine: biomarker testing for diagnosis and treatment of cardiovascular disease

**DOI:** 10.3389/fmed.2025.1564155

**Published:** 2025-02-27

**Authors:** Hendrianus Hendrianus, Eliano Navasere, Diana Gorog, Paul A. Gurbel, Sang-Wook Kim, Young-Hoon Jeong

**Affiliations:** ^1^Division of Cardiology, Chung-Ang University Gwangmyeong Hospital, Gwangmyeong, Republic of Korea; ^2^Division of Cardiology, Natuna General Hospital, Natuna, Indonesia; ^3^Department of Clinical and Interventional Cardiology, Sassari University Hospital, Sassari, Italy; ^4^Faculty of Medicine, National Heart and Lung Institute, Imperial College London, London, United Kingdom; ^5^Centre for Health Services Research, School of Life and Medical Sciences, University of Hertfordshire, Hatfield, United Kingdom; ^6^Sinai Center for Thrombosis Research and Drug Development, Sinai Hospital of Baltimore, Baltimore, MD, United States; ^7^Department of Internal Medicine, Chung-Ang University College of Medicine, Seoul, Republic of Korea; ^8^Chung-Ang University Thrombosis and Biomarker Center, Chung-Ang University Gwangmyeong Hospital, Gwangmyeong, Republic of Korea

**Keywords:** cardiovascular biomarkers, precision medicine, risk stratification, diagnostic biomarkers, therapeutic monitoring

## Introduction

Precision medicine has emerged as a transformative approach in healthcare, customizing diagnostic and therapeutic strategies to the unique characteristics of individual patients. In the context of cardiovascular disease (CVD), biomarker testing has become essential, providing critical insights for diagnosis, risk stratification, prognosis, monitoring, and treatment responses. Cardiovascular (CV) biomarkers are invaluable tools in this field, offering precise measurements of disease processes and enabling targeted therapeutic interventions. The studies highlighted in this Research Topic demonstrate advancements in CV biomarker research, emphasizing their role in enhancing precision medicine for CVD.

## Diagnostic biomarkers

Genetic biomarkers may hold promise, as demonstrated by Jaouadi et al., for revisiting the genetic underpinnings of hypertrophic cardiomyopathy (HCM). HCM, a condition fraught with diagnostic challenges, often leaves over half of patients without a precise genetic diagnosis. By reanalyzing exome sequencing data from the HYPERGEN cohort and incorporating newly identified HCM-associated genes such as SVIL, FHOD3, and TRIM63, the study achieved a 9% increase in variant identification of HCM. This dynamic approach to genetic biomarker research underscores its potential to enhance diagnostic accuracy and personalized care for HCM patients.

Another notable investigation, led by Martin-Virgala et al., explored immune cell biomarkers for diagnosing and monitoring vascular damage in patients with chronic kidney disease (CKD). Vascular calcification and atherosclerosis in CKD are often driven by chronic inflammation. By analyzing immune cell profiles in pre-dialysis CKD patients, the researchers identified elevated senescent T cells and altered Tang cells exhibiting immunosenescence, which strongly correlated with vascular stiffness as measured by pulse wave velocity (PWV). These findings position immune cell subsets as critical biomarkers, paving the way for early detection and targeted interventions in CKD-related CV complications.

One significant contribution comes from research on the Coronary Angiography-Based Index of Microcirculatory Resistance (CAG-IMR), introduced by Fan et al.. This novel diagnostic imaging biomarker addressed the presence of coronary microvascular dysfunction (CMD), a condition that is often difficult to diagnose with traditional wire-based methods due to their technical complexity and cost. CAG-IMR offers an efficient, wire-free alternative that has been validated against wire-based IMR in a cohort of 139 patients and 201 vessels. With a strong correlation (r = 0.84, *p* < 0.001) and high diagnostic performance (AUC = 0.97), it achieves a sensitivity of 92.7% and specificity of 95.6%. These findings highlight its potential to improve accessibility and precision in CMD diagnosis.

The clinical utility of vascular biomarkers is exemplified in a study by Kim et al., which explored prognostic implication of brachial-ankle PWV (baPWV) following percutaneous coronary intervention (PCI). Elevated baPWV, indicative of large arterial stiffness, was linked to higher risks of all-cause mortality, non-fatal myocardial infarction, and major bleeding over a 4-year follow-up. Patients with high baPWV exhibited significantly worse clinical outcomes, with adjusted hazard ratios ranging from 1.40 to 1.94. These findings establish baPWV as a vital prognostic vascular biomarker, aiding in risk stratification and guiding post-PCI management strategies.

## Therapeutic biomarkers

In the area of therapeutic monitoring, Lee et al. investigated pharmacodynamic biomarkers in the context of Mono-antiplatelet and colchicine-based therapy (MACT) vs. dual-antiplatelet therapy (DAPT) for acute coronary syndrome (ACS) patients undergoing PCI. The study revealed that colchicine significantly reduced significantly the level of high-sensitivity C-reactive protein (hs-CRP) at 1 month post-PCI compared to DAPT, with showing the similar level of platelet reactivity. This positions hs-CRP as a valuable pharmacodynamic biomarker for monitoring inflammatory responses, supporting the integration of anti-inflammatory strategies into ACS management.

## Future perspectives

The studies featured in this Research Topic highlight the diverse applications of CV biomarkers in precision medicine. Diagnostic biomarkers, such as the CAG-IMR index and immune cell subsets, facilitate early and accurate detection of CVD. Risk stratification and prognostic biomarkers, including baPWV, help identify high-risk individuals who may benefit from tailored interventions. Monitoring biomarkers, such as immune profiles in CKD, offer insights into disease progression and treatment efficacy. Pharmacodynamic biomarkers like hs-CRP guide therapeutic decisions, enhancing the personalization of CV care.

Building on these diverse applications, recent advancements in CV biomarker research have further expanded the role of precision medicine, deepening our understanding of the complex factors influencing heart diseases. However, integrating biomarkers into clinical practice presents several challenges, including the need for rigorous validation across diverse populations and the establishment of standardized protocols for their use (1). Future research should aim to address these challenges while expanding the applications of CV biomarkers. Multi-omics approaches, which integrate genomics, proteomics, and metabolomics, hold tremendous potential for discovering novel biomarkers and unraveling complex disease mechanisms (2). Furthermore, artificial intelligence and machine learning can accelerate biomarker discovery, optimize data interpretation, and facilitate clinical implementation, thus advancing the field of precision medicine (3). Additionally, emerging technologies, such as telemedicine and wearable devices, offer promising avenues for incorporating biomarkers into routine patient care, enhancing both precision and accessibility in CV health management (4).

## Conclusion

CV biomarker testing is a cornerstone of future precision medicine. The studies discussed in this Research Topic exemplify the transformative potential of CV biomarkers in improving patient care. By addressing existing challenges and embracing innovative research and technology, the medical community can further leverage the power of CV biomarkers to improve CV outcomes and shape the future of precision medicine.
